# Exploring motivational and contextual factors influencing medical career choice: a theory-informed study

**DOI:** 10.3389/fpsyg.2025.1722595

**Published:** 2025-12-10

**Authors:** Selcen Öncü

**Affiliations:** Department of Medical Education, Faculty of Medicine, Aydın Adnan Menderes University, Aydın, Türkiye

**Keywords:** medical career choice, motivation, self-efficacy, intrinsic and extrinsic values, social cognitive career theory, expectancy value theory, cultural influences, emotion in education

## Abstract

**Background:**

Career choice in medicine represents a multidimensional decision influenced by motivational, emotional, and sociocultural dynamics. Understanding how these factors interact is essential for developing educational environments that nurture sustained professional commitment. Drawing on the Social Cognitive Career Theory (SCCT) and the Expectancy–Value Theory (EVT), this study explored how self-efficacy beliefs, outcome expectations, task values, and contextual supports shape medical career motivation among first-year medical students.

**Methods:**

A cross-sectional quantitative design was used, involving 297 first-year medical students at a public university in western Türkiye. Data were collected online and anonymously during the 2022–2023 academic year, using a researcher-developed and pilot-validated questionnaire comprising 33 items assessing motivational and contextual determinants of career choice. Questionnaire items were structured according to the constructs of SCCT and EVT. Descriptive and inferential statistical analyses (t-tests, ANOVA, *p* < 0.05; SPSS v25) were performed to examine associations between motivational dimensions and demographic variables.

**Results:**

Students’ strongest motivations were intrinsic and self-efficacy–based—particularly personal desire to study medicine, belief in being a good physician, and moral satisfaction—corresponding to SCCT’s self-efficacy and EVT’s intrinsic value dimensions. Job security and professional prestige also received high ratings, reflecting positive outcome expectations and utility value. In contrast, environmental barriers (e.g., workload, media influence, and perceived low income) had comparatively lower influence. Female students reported higher intrinsic and altruistic motivations, whereas male students emphasized financial stability and outcome expectations. Parental education and age were also associated with higher efficacy and expectancy beliefs, aligning with SCCT’s view of environmental support as a reinforcing factor.

**Conclusion:**

The findings suggest that intrinsic motivation and perceived competence outweigh external constraints in shaping early medical career intentions. Framing medical career choice through SCCT and EVT offers a comprehensive understanding of how emotional, motivational, and cultural factors intersect in professional identity formation. These insights can inform interventions that strengthen self-efficacy, moral engagement, and contextual support in medical education.

## 1 Introduction

Career choice is a complex and multidimensional decision for students, influenced by cultural, economic, familial, and individual characteristics, as well as prior medical exposure and competencies. These factors vary across countries due to cultural and socioeconomic differences (Shahab et al., 2011; Tiwari et al., 2016; Goel et al., 2018). Medicine has long been one of the most prestigious and preferred professions, positioning physicians as key health providers.

As of 2024, Türkiye ranks among the leading countries worldwide with 116 medical faculties actively providing undergraduate education (Sistemi, 2025). Every year, more than two million students sit for the national university entrance examination, and only those within the highest percentile are able to secure admission to a state medical faculty. In this context, choosing a profession based not only on exam performance but also on personal interests and abilities becomes particularly challenging. Understanding students’ motivations and reasons for selecting medicine is therefore critical for training competent and motivated physicians. This study aims to explore the factors influencing first-year medical students’ choice of medical school.

Although numerous studies have explored the motives behind medical career choice (Goel et al., 2018; Clack and Head, 1998; Girasek et al., 2011) most have focused on students from Western or high-income contexts. There is limited evidence from Türkiye, where medical education is highly competitive, culturally diverse, and shaped by socioeconomic pressures. Understanding how motivational and contextual factors interact in this setting may provide valuable insights for curriculum development, student guidance, and workforce planning in medical education. Therefore, this study examines the topic within the framework of two complementary and well-established career development theories; Social Cognitive Career Theory (SCCT) (Lent et al., 1994) and Expectancy–Value Theory (EVT) (Wigfield and Eccles, 2000), to provide a comprehensive and theory-informed understanding of why and how students choose medicine as a career. SCCT suggests that self-efficacy beliefs and outcome expectations, in interaction with interests and environmental supports, shape career choices (Lent et al., 1994). EVT emphasizes that achievement-related choices are determined by individuals’ expectations of success and the subjective task value they attribute to an option (utility, importance, interest, and cost) (Wigfield and Eccles, 2000). In the Turkish context, rational considerations such as job security and income can also be explained through human capital and rational choice perspectives.

Guided by these theoretical frameworks, this study examines how students’ emphasis on factors such as personal desire, belief in becoming a good physician, high moral satisfaction, employment security, and financial benefits can be theoretically justified.

## 2 Materials and methods

### 2.1 Study design

This study was designed as a quantitative, descriptive, cross-sectional study to explore the factors influencing first-year medical students’ decisions to enrol in medical school. The study was conducted at the Faculty of Medicine, Aydın Adnan Menderes University, located in western Türkiye, during the 2022–2023 academic years.

### 2.2 Participants

The target population included all 310 first-year medical students actively attending classes during the study period. No sampling was performed, as the study aimed to reach the entire cohort. A total of 297 students (95.8%) voluntarily participated in the study by completing the online questionnaire. Prior to participation, all students were verbally informed about the voluntary nature and purpose of the study, and informed consent was obtained electronically. Participants were assured of anonymity and confidentiality of their responses.

### 2.3 Data collection tools

Data were collected using a structured questionnaire developed by the researchers based on the conceptual frameworks of the SCCT and EVT. The questionnaire was first evaluated by four expert medical educators and pilot-tested among 20 s-year medical students to ensure clarity and relevance. After revisions, the internal consistency reliability was confirmed with a Cronbach’s *α* of 0.864, indicating good reliability.

The questionnaire consisted of two main parts: Socio-demographic variables (13 items); including sex, age, type of high school, parental education, and place of residence. In Türkiye, Science High Schools and Anatolian High Schools are both selective secondary institutions; the former emphasizes intensive STEM education, while the latter offers a balanced curriculum combining sciences and humanities to prepare students for higher education (Supplementary material 1).

Motivational and contextual factors (33 items); including 22 items assessing positive motivations (“factors influencing enrolment”) and 11 items addressing hesitation or deterrent factors (hesitation/deterrent items were completed only by students who reported experiencing any hesitation when choosing medicine). All items were rated on a 5-point Likert scale (1 = least important/did not influence at all, 5 = most important/strongly influenced) (Supplementary material 1).

### 2.4 Data analysis

Statistical analyses were performed using IBM SPSS Statistics 25.0 software package. Descriptive statistics [frequency, percentage, mean, and standard deviation (SD)] were calculated. Percentages were based on the total sample (*N* = 297). All questionnaire items were rated on a 5-point Likert scale (0 = not influential, 5 = very influential), and mean scores were calculated based on this response range. Group comparisons were conducted using the Chi-square test, independent samples t-test, and one-way ANOVA. The significance level was set at *p* < 0.05.

### 2.5 Construct validity (exploratory factor analysis–methods)

Exploratory factor analysis (EFA) was conducted using Principal Component Analysis with Varimax rotation to assess construct validity. Only the 22 items completed by all participants (*N* = 297) were included in the analysis, whereas the 11 hesitation-specific items were completed only by a subgroup of students (*n* = 90) and were therefore not included in the factor analysis because they were not administered to the full sample. Sampling adequacy was assessed using the Kaiser–Meyer–Olkin (KMO) statistic, and factorability was examined via Bartlett’s test of Sphericity.

## 3 Results

### 3.1 Participant characteristics

A total of 297 first-year medical students participated in the study, of whom 50.2% (*n* = 149) were female and 49.8% (*n* = 148) were male. The mean age was 19.12 ± 1.33 years (range: 18–27). Additional socio-demographic characteristics of the participants are presented in Table 1.

**Table 1:** Demographic characteristics of the participants.

Characteristic	*N*	%
Gender—Female	149	50.2
Gender—Male	148	49.8
Mean age (years ± SD)	19.12 ± 1.33	-
High school type—Science High School	120	40.4
High school type—Anatolian High School	150	50.5
Other	27	9.1
Parental education—University or higher	98	33.0

Percentages are calculated based on valid responses (*N* = 297), SD = Standard Deviation.

### 3.2 Motivational and contextual factors

Table 2 presents the mean scores of items assessing motivational factors in relation to SCCT and EVT dimensions. Within the SCCT perspective, items related to *self-efficacy* (e.g., personal desire, belief in being a good physician) and *outcome expectations* (e.g., moral satisfaction, job security and professional prestige) received the highest mean scores. In contrast, items reflecting *environmental influences* and *cost-related barriers* (e.g., family health problems, peer or media effects) had the lowest mean ratings (Table 2).

**Table 2:** Mean scores of questionnaire items mapped to SCCT and EVT theoretical dimensions.

Items	%	Mean ± SD	SCCT dimension	EVT dimension
Personal desire to study medicine	76.8	4.05 ± 1.06	Self-efficacy	Intrinsic value
Belief in being a good physician	71.1	3.91 ± 1.15	Self-efficacy	Expectancy
High moral or emotional satisfaction derived from the medical profession	70.4	3.89 ± 1.19	Outcome expectations	Intrinsic value
Perceived employment security in medicine	69.0	3.77 ± 1.10	Outcome expectations	Utility value
Perception of medicine as a respected profession	64.6	3.75 ± 1.17	Outcome expectations	Social value
Perceived personality fit with the medical profession	58.9	3.61 ± 1.23	Self-efficacy	Intrinsic value
Altruistic motivation and willingness to help others	58.6	3.57 ± 1.23	Outcome expectations	Intrinsic value
Influence of university entrance exam score	61.0	3.56 ± 1.24	Environmental factor	Cost/external expectancy
Expectation of opportunities to work abroad	57.2	3.46 ± 1.34	Outcome expectations	Utility value
Family influence on the choice of medicine	55.3	3.44 ± 1.13	Environmental support/barrier	Extrinsic value
Perception of medicine as a suitable field for scientific research	53.8	3.43 ± 1.29	Goal setting/choice action	Utility value
Identification of medicine as an ideal profession	51.5	3.41 ± 1.40	Goal/self-efficacy	Intrinsic value
Expectation of favourable financial income	51.5	3.34 ± 1.11	Outcome expectations	Extrinsic value
Perception of medicine as a suitable field for an academic career	50.5	3.31 ± 1.29	Goal setting	Utility value
Interest in biology courses during secondary education	42.4	3.18 ± 1.33	Learning experience	Expectancy
Influence of peers and social environment	24.0	2.59 ± 1.16	Environmental influence	External expectancy
Influence of teachers or mentors	23.2	2.56 ± 1.15	Environmental influence	External expectancy
Absence of physicians within the family	28.3	2.43 ± 1.40	Environmental barrier	Cost
Experience of health problems in family members or self	20.5	2.30 ± 1.26	Environmental influence	External expectancy
Impact of media and social media representations	16.9	2.23 ± 1.15	Environmental influence	External expectancy
Presence of healthcare professionals in the family	16.5	2.01 ± 1.24	Environmental influence	External expectancy
Positive impact of the COVID-19 pandemic	7.8	2.00 ± 1.02	Environmental barrier	External expectancy

Percentages (%) indicate the proportion of students who rated each item as “4” or “5” on the 5-point Likert scale. Percentages are calculated based on the total sample (*N* = 297). Mean values reflect responses on a 5-point Likert scale (1 = not influential at all, 5 = very influential). SD = standard deviation. Each questionnaire item represents a motivational statement mapped to relevant constructs of SCCT and EVT to illustrate the theoretical alignment of findings.

Within the EVT perspective, *intrinsic value* components such as altruism and moral satisfaction were the most influential, followed by *utility and extrinsic values* related to job stability and income. These findings indicate that students’ career motivations are primarily driven by *intrinsic motivation, self-efficacy beliefs, and positive outcome expectations*, rather than by external pressures or contextual determinants (Table 2).

Several barriers were also identified, the most prominent were *inadequate income*, challenging working conditions, and decreased societal respect for the profession. These findings reflect perceptions reported only by the subgroup of students who experienced hesitation when choosing medicine (*n* = 90). These factors correspond to the environmental barrier and cost constructs described in the SCCT and EVT frameworks (Table 3).

**Table 3:** Factors deterring/hesitating participants from choosing the medical profession (mapped to SCCT and EVT dimensions).

Items	%	Mean ± SD	SCCT dimension	EVT dimension
Inadequate income for physicians	72.1	3.81 ± 1.37	Environmental barrier/outcome expectation	Cost/utility value
Unsuitable or difficult working conditions for physicians	65.2	3.60 ± 1.37	Environmental barrier	Cost
Decreased societal respect for physicians	60.5	3.47 ± 1.37	Environmental influence	Social value (negative)
Increasing violence against physicians	44.9	3.05 ± 1.48	Environmental barrier	Cost
Negative news and reports in media and social media	41.9	3.07 ± 1.45	Environmental influence	External expectancy
Lengthy duration of medical education	44.2	3.00 ± 1.40	Learning experience/environmental barrier	Cost
Difficulty of medical education	37.2	2.93 ± 1.30	Self-efficacy (challenge perception)	Cost
Compulsory public service	34.9	2.91 ± 1.38	Environmental barrier	Cost/external expectancy
Increasing physician migration abroad	34.9	2.79 ± 1.36	Outcome expectation (negative)	Cost/Utility
Negative impact of the COVID-19 pandemic on physicians	32.6	2.72 ± 1.45	Environmental influence	External expectancy
The high cost of medical education	30.2	2.67 ± 1.43	Environmental barrier	Cost

This table presents deterring/ hesitation factors perceived by first-year medical students, conceptually mapped to the components of SCCT and EVT. Percentages (%) indicate the proportion of students who rated each item as “4” or “5” on the 5-point Likert scale. and are calculated based on the subgroup of students who completed the hesitation items (*n* = 90). Mean values reflect responses on the same Likert scale (0 = not influential, 5 = very influential). SD = standard deviation.

### 3.3 Construct validity results

Exploratory factor analysis demonstrated strong sampling adequacy (KMO = 0.852) and a significant Bartlett’s test of Sphericity, χ^2^ (231) = 2830.14, *p* < 0.001. Five components with eigenvalues greater than 1 were extracted, explaining 61.6% of the total variance. The rotated factor loadings, presented in Supplementary material 2, showed a clear and interpretable structure. The five components corresponded conceptually to intrinsic–academic motivation, contextual–social influences, deterrent perceptions, workload and practice-related concerns, and prestige–financial expectations (Supplementary material 2).

Overall, the factor solution demonstrated strong construct validity and coherent alignment with SCCT and EVT.

### 3.4 Comparison of SCCT and EVT dimensions by demographic variables

When SCCT and EVT dimension scores were compared according to demographic variables, several significant differences emerged. Mother’s education: Students, whose mothers had higher education levels scored significantly higher in self-efficacy (*p* = 0.042) and expectancy (*p* = 0.038) than those whose mothers had lower education levels. Father’s education: Higher paternal education was associated with significantly higher Value scores (*p* = 0.029). Age group: students aged 20 years and above reported significantly higher Outcome expectations (*p* = 0.016) compared to those aged 19 years or younger.

These demographic effects align with SCCT assumptions that environmental and familial support systems reinforce self-efficacy and expectancy beliefs, while maturity and age-related experience enhance outcome expectations in career decision-making.

### 3.5 Gender differences

Statistically significant gender differences were observed in two motivational domains. Female students reported higher scores for personal factors such as *desire to study medicine* and *moral satisfaction* (4.70 ± 0.55 vs. 4.50 ± 0.65, *p = 0.02*), *reflecting stronger self-efficacy* and *intrinsic value* dimensions within the SCCT and EVT frameworks. In contrast, male students assigned greater importance to expectation of favourable income (4.60 ± 0.65 vs. 4.30 ± 0.70, *p = 0.03*), consistent with higher extrinsic task value and outcome expectations in EVT.

These findings suggest that female students are more driven by internal satisfaction and confidence in their abilities, whereas male students are more motivated by financial and instrumental rewards. Such gendered motivational orientations align with previous evidence that cultural and socioeconomic contexts shape the relative centrality of altruistic versus pragmatic motives in medical career choice.

### 3.6 Integration of findings with theoretical frameworks

Table 4 presents the alignment between the study findings and the components of the SCCT, which was used as a guiding framework. Self-efficacy-related items included students’ belief in becoming good physicians, their own desire, and perceived personal suitability for the profession. Outcome expectations, both positive and negative, encompassed job security, societal respect, income expectations, as well as concerns about low remuneration, poor working conditions, and workplace violence. Personal goals were reflected in high moral satisfaction, interest in scientific research, and willingness to help people. Environmental/contextual factors included gender differences, parental education, and the presence of healthcare workers in the family.

**Table 4:** Alignment of study findings with components of the SCCT.

SCCT component	Key findings from current study	Illustrative items
Self-efficacy	Confidence in ability to become a good physician and perceived personal suitability for medicine.	“Belief in being a good physician,” “Personal desire,” “Personality fit.”
Outcome expectations	Positive expectations about job security, respect, and financial stability; awareness of low remuneration and workload barriers.	“Job security,” “High moral satisfaction,” “Favourable income,” “Unfavourable working conditions.”
Goals/choice actions	Aspirations toward moral fulfilment, altruism, and academic or research careers.	“Helping others,” “Scientific research interest,” “Academic career preference.”
Environmental/contextual influences	Effects of parental education, gender, and socioeconomic background; limited influence of peers or media.	“Parental education,” “Gender differences,” “Media influence.”

This table summarizes the conceptual mapping between empirical findings and the major constructs of SCCT, serving as a framework for interpreting results.

Table 4 illustrates the alignment between the key components of SCCT and the findings from the current study on factors influencing first-year medical students’ choice of medicine as a career.

## 4 Discussion

This study explored the motivational and contextual factors influencing medical students’ choice of medicine through the SCCT and EVT frameworks. According to SCCT, career interests and decisions are shaped by self-efficacy and outcome expectations within supportive or constraining environments (Lent et al., 1994). Similarly, EVT proposes that the subjective value attached to a career option and the expectation of success determine choice behavior (Eccles and Wigfield, 2002). These results align with prior international literature emphasizing the predictive power of intrinsic motivation and perceived competence in medical career choice (Wang et al., 2022; Chiu et al., 2023; Sobral, 2004).

### 4.1 Personal and intrinsic motivations

The most core motivational themes—personal desire to study medicine, belief in being a good physician, and moral satisfaction—reflect the intersection of self-efficacy (SCCT) and intrinsic value (EVT). Similar to Bansal and Pagidas (2025), who reported that intrinsic motivation and perceived competence predict sustained engagement and academic performance, this theoretical perspective suggests that intrinsic goals tend to play a more influential role than external rewards or constraints in shaping early medical career interests. In Türkiye, where admission to medical faculties is highly competitive, students’ strong entrance-exam performance may reinforce their sense of competence and autonomy, further strengthening self-efficacy and professional commitment.

As prior research has shown, SCCT provides a robust framework linking self-efficacy, outcome expectations and contextual supports to career choices (Lent et al., 1994; Chiu et al., 2023; Sheu et al., 2010). Consistent with this framework, the pattern observed in our study suggests that positive self-beliefs and meaningful task value may buffer the impact of perceived barriers during the early stages of career formation.

Recent studies from Europe similarly highlight the multidimensional nature of medical career motivation. Montenegrin students emphasized intrinsic values and professional ideals as key determinants of choosing medicine (Zvrko et al., 2024), while Portuguese findings showed that intrinsic motivation strengthens as students advance academically and develop a clearer professional identity (Matos Sousa et al., 2025). German and Polish studies further illustrated how contextual factors—such as working conditions, lifestyle expectations, and prestige—interact with stable intrinsic motivations in shaping career intentions (Leutritz et al., 2024; Sarnowska et al., 2025). Overall, these findings indicate that the motivational patterns observed in our study are consistent with emerging trends across European settings, where intrinsic aspirations and contextual influences jointly guide early medical career decisions.

### 4.2 Sociocultural and demographic determinants

Sociocultural background and family education are well-recognized contextual determinants within SCCT, as parental education, social capital and academic support shape students’ self-efficacy and career expectations. In many settings, students from academically advantaged families benefit from clearer guidance and stronger confidence in their abilities, whereas those from less educated backgrounds tend to rely more on external supports such as teachers, peers, or institutional mentoring systems (Odusanya et al., 2000). These dynamics highlight the importance of considering both familial and environmental resources when interpreting early career motivation in medical students. Developmental factors may also play a role, as maturing students often demonstrate more crystallized expectations and clearer career reasoning, consistent with broader developmental and motivational research.

Gender-related motivational tendencies reported in prior research suggest that broader social role expectations and value orientations may shape how male and female students engage with the medical profession (Heiligers, 2012; Vaglum and Ekeberg, 1999). Studies, including the recent work by Saxena et al. (2024), indicate that gendered patterns in learning strategies, altruistic values, and career priorities continue to influence professional identity development in medical education. These longstanding cross-cultural trends highlight the importance of considering gender as a sociocultural factor that interacts with intrinsic motivation, perceived rewards, and contextual expectations when interpreting early career intentions (Saxena et al., 2024). These differences may reflect gendered social roles and expectations around care and responsibility, as noted in broader SCCT applications (Wang et al., 2022). From a curriculum-development perspective, recognizing these patterns can help educators design interventions that balance humanistic and pragmatic motives and promote equitable participation.

### 4.3 Perceived barriers and emerging challenges

Research on early career choice frequently notes that pragmatic concerns; such as income, workload or societal recognition, tend to have limited centrality during the initial stages of medical identity formation. The relatively limited centrality of these barriers at entry may indicate that strong vocational ideals overshadow pragmatic concerns, a phenomenon described in literature on early medical identity formation. This finding suggests that while students recognize barriers, they are not significantly deterred by them at the entry stage. Comparable results have been reported in studies where early-stage medical students idealized the profession before encountering systemic pressures (Tiwari et al., 2016; Girasek et al., 2011). The minimal reported influence of the COVID-19 pandemic in our cohort aligns with recent evidence that the symbolic and moral value of medicine continues to outweigh pragmatic concerns. While some studies conducted during and after the pandemic documented increased hesitation among medical and premedical students due to perceived occupational risks, disrupted training, and emotional fatigue (Nishimura et al., 2024; Doan et al., 2023), others have emphasized that the pandemic also strengthened students’ moral identification with the profession (Joseph et al., 2024).

### 4.4 Implications for medical education

Understanding motivational and contextual dynamics through SCCT and EVT provides actionable insights for curriculum development. Educational interventions that enhance self-efficacy, foster reflective and moral growth, and strengthen professional identity can help sustain intrinsic motivation throughout medical training. Structured mentorship, early clinical exposure, and positive role modeling may serve as protective factors against the motivational decline often observed in later academic years (Bansal and Pagidas, 2025). At the institutional level, addressing workload, safety and compensation-related concerns remains essential to align students’ idealistic aspirations with realistic career expectations and ensure long-term professional fulfillment.

### 4.5 Limitations and future directions

This single-centre, cross-sectional study limits generalizability, and the reliance on self-reported data may reflect perceptions rather than actual behaviors. Additionally, responses may have been influenced by social desirability bias, as students might have tended to report motivations that appear professionally or socially acceptable. Future multi-institutional and longitudinal studies should explore how motivational factors evolve throughout medical training. Complementary qualitative approaches could also provide deeper insight into how individual, familial, and societal influences interact within SCCT and EVT frameworks to shape sustained professional identity and career satisfaction.

### 4.6 Implications for practice

The findings highlight the need for structured early clinical exposure and meaningful contact with role models to strengthen students’ self-efficacy and clarify their expectations regarding the medical profession. Integrating professional identity formation activities—such as reflective writing, narrative medicine, and medical humanities sessions—into the early curriculum may further support students’ intrinsic motivation and sense of belonging. Additionally, programs that introduce the diverse career pathways within medicine, combined with structured career guidance, can help students develop more realistic and sustainable motivational profiles. Collectively, these educational strategies align with SCCT and EVT frameworks by enhancing students’ perceived competence, clarifying outcome expectations, and reinforcing the personal and professional values that shape long-term career satisfaction.

## 5 Conclusion

In conclusion, this theory-informed study contributes to a deeper understanding of how motivational and contextual determinants shape medical students’ career choices. Integrating Social Cognitive Career Theory and Expectancy–Value Theory provides a coherent conceptual framework for interpreting medical career motivation and for guiding educational strategies that foster sustained engagement and long-term professional commitment.

## Data Availability

The raw data supporting the conclusions of this article will be made available by the authors, without undue reservation.

## References

[ref1] Bansal S. Pagidas K. (2025). Strength of motivation and academic performance of medical students: a longitudinal study. BMC Med. Educ. 25:1154. doi: 10.1186/s12909-025-07733-3, 40781667 PMC12333129

[ref2] Chiu H. Y. Chiang C. M. Kang Y. N. Chen C. C. Wu C. C. Chiu Y. H. . (2023). Development of a social cognitive career theory scale for measuring the intention to select surgery as a career. Heliyon 9:e21685. doi: 10.1016/j.heliyon.2023.e21685, 38027609 PMC10665719

[ref3] Clack G. B. Head J. O. (1998). Why do students want to do medicine? Med. Educ. 32, 219–220. doi: 10.1046/j.1365-2923.1998.0188o.x

[ref4] Doan L. P. Dam V. A. T. Boyer L. Auquier P. Fond G. Tran B. . (2023). Impacts of COVID-19 on career choices in health professionals and medical students. BMC Med. Educ. 23:387. doi: 10.1186/s12909-023-04328-8, 37237404 PMC10215048

[ref5] Eccles J. S. Wigfield A. (2002). Motivational beliefs, values, and goals. Annu. Rev. Psychol. 53, 109–132. doi: 10.1146/annurev.psych.53.100901.135153, 11752481

[ref6] Girasek E. Molnár R. Eke E. Szócska M. (2011). The medical career choice motivations—results from a Hungarian study. Cent. Eur. J. Med. 6, 502–509. doi: 10.2478/s11536-011-0034-0, 40909103

[ref7] Goel S. Angeli F. Dhirar N. Singla N. Ruwaard D. (2018). What motivates medical students to select medical studies: a systematic literature review. BMC Med. Educ. 18:16. doi: 10.1186/s12909-018-1123-4, 29343262 PMC5772649

[ref8] Heiligers P. J. M. (2012). Gender differences in medical students’ motives and career choice. BMC Med. Educ. 12:82. doi: 10.1186/1472-6920-12-82, 22913471 PMC3575371

[ref9] Joseph N. Satapathy A. Singh V. Palia A. Bansal P. Verma A. K. . (2024). Career perceptions during the COVID-19 pandemic among undergraduate medical students and interns in South India. Int. J. Acad. Med. 10, 26–34. doi: 10.4103/ijam.ijam_67_23

[ref10] Lent R. W. Brown S. D. Hackett G. (1994). Toward a unifying social cognitive theory of career and academic interest, choice, and performance. J. Vocat. Behav. 45, 79–122. doi: 10.1006/jvbe.1994.1027

[ref11] Leutritz T. Krauthausen M. Simmenroth A. König S. (2024). Factors associated with medical students’ career choice in different specialties: a multiple cross-sectional questionnaire study at a German medical school. BMC Med. Educ. 24:798. doi: 10.1186/s12909-024-05751-1, 39049024 PMC11270969

[ref12] Matos Sousa R. Santos M. Marangoni M. Pereira V. H. (2025). Motivation in medical students: higher intrinsic motivation among graduate-entry students across academic stages. Front. Med. 12:1625352. doi: 10.3389/fmed.2025.1625352, 40636376 PMC12238096

[ref13] Nishimura A. Miyoshi T. Otsuka F. Matsukawa A. (2024). Influence of the coronavirus disease 2019 pandemic on the post-graduate career paths of medical students: a cross-sectional study. BMC Med. Educ. 24:55. doi: 10.1186/s12909-023-05021-6, 38200534 PMC10782557

[ref14] Odusanya O. Alakija W. Akesode F. (2000). Socio-demographic profile and career aspirations of medical students in a new medical school. Nigerian Postgrad. Med. J. 7, 112–115, 11257915

[ref15] Sarnowska P. Terech J. Bikowska K. Guziak M. Walkiewicz M. (2025). Assessing the impact of medical studies on students’ motivation, satisfaction, stress and values in Poland: a cross-sectional study. BMC Med. Educ. 25:714. doi: 10.1186/s12909-025-07287-4, 40380129 PMC12085074

[ref16] Saxena S. Wright W. S. Khalil M. K. (2024). Gender differences in learning and study strategies impact medical students’ preclinical and USMLE step 1 examination performance. BMC Med. Educ. 24:504. doi: 10.1186/s12909-024-05494-z, 38714975 PMC11077801

[ref17] Shahab F. Hussain H. Inayat A. Shahab A. (2011). Attitudes of medical students towards their career—perspective from Khyber-Pukhtunkhwa. Medicine 63, 1017–1020.

[ref18] Sheu H.-B. Lent R. W. Brown S. D. Miller M. J. Hennessy K. D. Duffy R. D. (2010). Testing the choice model of social cognitive career theory across Holland themes: a meta-analytic path analysis. J. Vocat. Behav. 76, 252–264. doi: 10.1016/j.jvb.2009.10.015

[ref19] Sistemi Yükseköğretim Bilgi Yönetim. (2025). Öğrenci İstatistikleri. Available online at: https://istatistik.yok.gov.tr

[ref20] Sobral D. T. (2004). What kind of motivation drives medical students’ learning quests? Med. Educ. 38, 950–957. doi: 10.1111/j.1365-2929.2004.01913.x, 15327676

[ref21] Tiwari R. Jain V. Arya R. Dwivedi S. Shrivastava D. Tiwari S. (2016). A study to assess the perceptions of first-year medical students for choosing medical school as a career. Int. J. Res. Med. Sci. 4, 2649–2655. doi: 10.18203/2320-6012.ijrms20161926, 27125159

[ref22] Vaglum P. Ekeberg Ø. (1999). Motivation for medical school: the relationship to gender and specialty preferences in a nationwide sample. Med. Educ. 33, 236–242. doi: 10.1046/j.1365-2923.1999.00293.x, 10336753

[ref23] Wang D. Liu X. Deng H. (2022). The perspectives of social cognitive career theory approach in current times. Front. Psychol. 13:1023994. doi: 10.3389/fpsyg.2022.1023994, 36533045 PMC9749854

[ref24] Wigfield A. Eccles J. S. (2000). Expectancy–value theory of achievement motivation. Contemp. Educ. Psychol. 25, 68–81. doi: 10.1006/ceps.1999.1015, 10620382

[ref25] Zvrko E. Popović N. Radunović M. Nikolić G. (2024). Motivational factors influencing the choice of medical studies and future career plans among Montenegrin students. Slovenian J. Public Health 63, 132–141. doi: 10.2478/sjph-2024-0018, 38881636 PMC11178028

