# Supplementary material for: Exploring motivational and contextual factors influencing medical career choice: a theory-informed study
Source: Front Psychol. 2025 Dec 10;16:1722595. doi: 10.3389/fpsyg.2025.1722595 (PMC12727634; doi:10.3389/fpsyg.2025.1722595)
Supplement: Supplementary file 1 [file Supplementary_file_1.docx]

**Supplementary File - 1**

**English Version of the Questionnaire**

*This supplementary file contains the full English version of the questionnaire used in the study, including demographic items, motivational factors, and deterrent/hesitation factors.*

**Section 1- Demographic Information**

**1. Your age:** ___ (write-in)

**2. Your gender:** ( ) Female ( ) Male ( ) Other / I don’t want to mention

**3. Type of high school you graduated from:**

a. Science High School

b. Anatolian High School

c. Basic High School

d. Private High School

e. High School Abroad

**4. Is this your first year at university?**

a. Yes b. No, I transferred from another university c. No, I am repeating the year

**5. How would you describe your family’s economic status?**

a. Low b. Moderate c. Good d. Very good

**6. The region in which you lived longest before starting university:**

a**.** Mediterranean

b. Eastern Anatolia

c. Aegean

d. South-eastern Anatolia

e. Central Anatolia

f. Black Sea

g. Marmara

h. Abroad

**7. Type of residential area you lived in before starting university:**

a. Village b. Town c. City

**8. Mother’s educational level:**

a. Primary school or below

b. Middle school

c. High school

d. University degree

e. Master’s/Doctoral degree

**9. Father’s educational level:**

a. Primary school or below

b. Middle school

c. High school

d. University degree

e. Master’s/Doctoral degree

**10. Is there a healthcare professional in your family?**

a. No b. Yes, my mother and/or father c. Yes, my sibling d. Yes, a close relative

**11. What was your ideal (dream) profession?**

a. Medicine b. A profession other than medicine

**12. If all professions offered the same job opportunities and working conditions, would you still choose medicine?**

a. Yes b. No

**13. Which statement best describes your university entrance preference?**

a. Medicine was my first-choice program b. Medicine was not my first choice

**Section 2- Motivational and Hesitation Factors**

| **2 A. Motivational Factors (22 items)**  *Please complete this section to indicate the factors that encouraged /motivated you to choose medicine.* | | | | | |
| --- | --- | --- | --- | --- | --- |
| *(Likert scale: 1 = Not influential at all, 5 = Very influential)* | **1** | **2** | **3** | **4** | **5** |
| 1. My own personal desire to study medicine |  |  |  |  |  |
| 1. My family’s influence |  |  |  |  |  |
| 1. Influence of my peers and social environment |  |  |  |  |  |
| 1. Influence of my teachers or mentors |  |  |  |  |  |
| 1. Influence of media and social media representations |  |  |  |  |  |
| 1. Presence of healthcare professionals in my family |  |  |  |  |  |
| 1. Absence of physicians within my family |  |  |  |  |  |
| 1. Experience of health problems in myself or family members |  |  |  |  |  |
| 1. Being a physician is the ideal profession in my mind |  |  |  |  |  |
| 1. My personality is suitable for the medical profession |  |  |  |  |  |
| 1. I like people and want to help others |  |  |  |  |  |
| 1. I believe that I can become a good physician |  |  |  |  |  |
| 1. My university entrance exam score |  |  |  |  |  |
| 1. My interest in biology courses during secondary education |  |  |  |  |  |
| 1. Medicine is a respected profession in society |  |  |  |  |  |
| 1. Medicine offers high moral or emotional satisfaction |  |  |  |  |  |
| 1. Expectation of favourable financial income offered by medicine |  |  |  |  |  |
| 1. Job security associated with medicine |  |  |  |  |  |
| 1. The positive impact of the COVID-19 pandemic |  |  |  |  |  |
| 1. Medicine is suitable field for an academic career |  |  |  |  |  |
| 1. Opportunities to work abroad |  |  |  |  |  |
| 1. Medicine is a suitable field for scientific research |  |  |  |  |  |

| **2 B. Hesitation Factors (11 items)**  *Please complete this section* ***only if you experienced any hesitation*** *in your decision to choose medicine.* | | | | | |
| --- | --- | --- | --- | --- | --- |
| (Likert scale: 1 = Not influential at all, 5 = Very influential) | **1** | **2** | **3** | **4** | **5** |
| 1. **Increasing violence against physicians** |  |  |  |  |  |
| 1. **Decreased societal respect for physicians** |  |  |  |  |  |
| 1. **Unsuitable or difficult working conditions for physicians** |  |  |  |  |  |
| 1. **Physicians do not receive adequate compensation for their work** |  |  |  |  |  |
| 1. **Compulsory public service for physicians** |  |  |  |  |  |
| 1. **Increasing migration of physicians to other countries** |  |  |  |  |  |
| 1. **Negative effects of the COVID-19 pandemic on physicians** |  |  |  |  |  |
| 1. **Medical education is academically demanding** |  |  |  |  |  |
| 1. **Medical education takes a long time to complete** |  |  |  |  |  |
| 1. **The high cost of medical education** |  |  |  |  |  |
| 1. **Negative news and reports in the media and social media** |  |  |  |  |  |
