# Supplementary material for: Exploring motivational and contextual factors influencing medical career choice: a theory-informed study
Source: Front Psychol. 2025 Dec 10;16:1722595. doi: 10.3389/fpsyg.2025.1722595 (PMC12727634; doi:10.3389/fpsyg.2025.1722595)
Supplement: Supplementary file 2 [file Supplementary_file_2.docx]

**Supplementary File - 2**

**Table 1 -** Rotated factor loadings for the 22-item motivation scale (Varimax rotation)

| **Item No** | **Factor 1** | **Factor 2** | **Factor 3** | **Factor 4** | **Factor 5** |  |
| --- | --- | --- | --- | --- | --- | --- |
| 1 | 0.721 | 0.006 | 0.333 | -0.108 | 0.014 |  |
| 2 | 0.008 | 0.533 | -0.034 | 0.468 | 0.026 |  |
| 3 | -0.048 | 0.446 | -0.030 | 0.502 | 0.419 |  |
| 4 | -0.105 | 0.349 | 0.089 | 0.526 | 0.465 |  |
| 5 | 0.124 | 0.210 | 0.056 | 0.159 | 0.740 |  |
| 6 | 0.045 | -0.003 | 0.029 | -0.112 | 0.756 |  |
| 7 | 0.220 | 0.189 | 0.076 | 0.710 | -0.140 | |
| 8 | 0.115 | -0.077 | 0.199 | 0.645 | 0.154 | |
| 9 | 0.798 | -0.118 | 0.180 | 0.082 | 0.115 | |
| 10 | 0.819 | 0.007 | 0.169 | 0.058 | 0.041 | |
| 11 | 0.769 | 0.026 | -0.017 | 0.260 | 0.038 | |
| 12 | 0.812 | 0.078 | 0.198 | 0.077 | 0.017 | |
| 13 | -0.039 | 0.691 | 0.113 | 0.167 | 0.039 | |
| 14 | 0.465 | 0.204 | 0.334 | -0.026 | 0.030 | |
| 15 | 0.622 | 0.535 | 0.054 | 0.045 | 0.119 | |
| 16 | 0.733 | 0.337 | 0.047 | 0.061 | 0.081 | |
| 17 | 0.214 | 0.613 | 0.440 | -0.146 | 0.114 | |
| 18 | 0.236 | 0.773 | 0.194 | 0.044 | 0.051 | |
| 19 | 0.220 | -0.137 | 0.309 | 0.284 | 0.465 | |
| 20 | 0.259 | 0.107 | 0.743 | 0.172 | 0.043 | |
| 21 | 0.085 | 0.282 | 0.689 | 0.114 | 0.133 | |
| 22 | 0.274 | 0.053 | 0.800 | 0.069 | 0.027 | |
| ***Note.*** *Loadings < 0.40 are suppressed for clarity. Extraction method: Principal Component Analysis. Rotation method: Varimax with Kaiser normalization.* | | | | | | |
